# Retraction: MicroRNA-367 directly targets PIK3R3 to inhibit proliferation and invasion of oral carcinoma cells

**DOI:** 10.1042/BSR-2019-3867_RET

**Published:** 2022-11-11

**Keywords:** Invasion, MicroRNA-367, Oral squamous cell carcinoma, PIK3R3, Proliferation

This article is being retracted from *Bioscience Reports* at the request of the authors, who found miscalculations in their raw data and are therefore concerned about the results and conclusions. It also follows receipt of a notification from a reader, alerting the Editorial Office to similarities between the Figure 3A panels of this paper and those from other articles. These other papers do not share any common authors, and include Figure 2B from Shi et al. 2018 (doi: 10.1186/s11658-018-0094-0), Figure 3A from Wang et al. 2020 (doi: 10.1242/bio.049478), and Figure 3A from Qui et al. 2018 (doi: 10.3892/ijo.2018.4483). The authors wish to retract the article. The Editor-in-Chief and Editorial Board agree with the retraction.

